# Post-stroke functional independence after brain stimulation: meta-analysis

**DOI:** 10.1590/1806-9282.20260935

**Published:** 2026-11-09

**Authors:** Döne Günay, Sevda Uzun

**Affiliations:** 1 Sivas Provincial Directorate of Health – Sivas, Turkey.; 2 Kırşehir Ahi Evran University, Faculty of Health Sciences, Department of Nursing, Department of Psychiatric Nursing – Kırşehir, Turkey.

**Keywords:** Activities of daily living, Meta-analysis, Rehabilitation, Stroke, Transcranial direct current stimulation

## Abstract

**OBJECTIVE:**

The aim of this study was to evaluate the effects of transcranial direct current stimulation on motor functional independence in patients with stroke.

**METHODS:**

A comprehensive literature search was conducted in PubMed, Web of Science, Cochrane Library, Google Scholar, and the Council of Higher Education (YÖK) Thesis Center through January 2026 without time restrictions. Eligible studies were identified according to predefined inclusion and exclusion criteria. Data from the included studies were synthesized using meta-analytic and narrative approaches.

**RESULTS:**

Eleven studies were included in the meta-analysis. The findings demonstrated that transcranial direct current stimulation significantly improved motor functional independence in patients with stroke (SMD=-3.198; 95%CI -4.215 to -2.180; Z=-6.158; p<0.001). Substantial heterogeneity was observed among the studies (I^2^=89.18%).

**CONCLUSION:**

The findings suggest that transcranial direct current stimulation may contribute to improvements in motor functional independence following stroke. However, further studies with standardized intervention protocols and larger sample sizes are needed to confirm these findings.

## INTRODUCTION

Stroke is a leading cause of mortality and long-term disability worldwide, often resulting in persistent neurological deficits that impair motor function and reduce independence in activities of daily living (ADL)^1^. Restoring motor functional independence is therefore a primary goal of stroke rehabilitation.

Transcranial direct current stimulation (tDCS) has emerged as a promising non-invasive neuromodulation technique in neurorehabilitation. By modulating cortical excitability, enhancing neuroplasticity, and restoring interhemispheric balance, tDCS may facilitate motor recovery following stroke^2,3^.

Numerous randomized controlled trials and systematic reviews have investigated the effects of tDCS and other non-invasive brain stimulation approaches on post-stroke motor outcomes, with many reporting potential benefits for motor recovery^4,5^. However, considerable heterogeneity in stimulation protocols, treatment duration, frequency, and outcome measures limits the comparability of findings^6,7^.

Beyond improvements in motor performance, patient-centered outcomes such as ADL and functional independence are essential indicators of rehabilitation success. Evidence regarding the effects of tDCS on these outcomes remains limited and inconsistent^8,9^. Therefore, this systematic review and meta-analysis aimed to evaluate the effects of tDCS on motor functional independence in patients with stroke.

## METHODS

This study was conducted as a systematic review and meta-analysis in accordance with the PRISMA (Preferred Reporting Items for Systematic Reviews and Meta-Analyses) guidelines^10^. To minimize the risk of bias, the processes of literature search, study selection, and data extraction were performed independently by two researchers. Subsequently, all stages were cross-checked by both researchers. The quality assessment of the studies included in the systematic review and meta-analysis was also conducted by the researchers.

### Inclusion criteria

In this study, the selection of studies was based on the PICOS framework:

- Population (P): Individuals with stroke
- Intervention (I): Application of tDCS
- Comparison (C): No application of tDCS
- Outcomes (O): Functional independence
- Study Design (S): Experimental and quasi-experimental studies published in Turkish or English

### Exclusion criteria

Studies in the form of letters to the editor, case-control studies, systematic reviews, and traditional (narrative) reviews were excluded from the scope of this research.

### Search strategy

A comprehensive literature search was conducted through January 2026 using PubMed, Cochrane Library, Web of Science, the Council of Higher Education (YÖK) Thesis Center, and Google Scholar. Medical Subject Headings-compatible keywords and their combinations were used, including "stroke," "transcranial direct current stimulation," "motor functional independence," and "activities of daily living." No time restrictions were applied because of the limited number of relevant studies.

### Study selection

Studies were selected according to the predefined inclusion and exclusion criteria. After duplicate removal and title/abstract screening, eligible full-text articles were assessed for inclusion. Ultimately, 11 studies were included in the meta-analysis.

### Data extraction

Two researchers independently extracted data using a standardized form that included study characteristics, intervention details, outcome measures, and main findings. Extracted data are summarized in Table 1.

**Table 1:** Characteristics and main findings of the studies included in the systematic review and meta-analysis.

Author	Study design	Measurement tools	Sample size	Intervention duration	Main findings	Quality score
Hamoudi et al.^18^	Randomized controlled trial	Fugl-Meyer Assessment (FMA)	Experimental: 18; control: 18	20 min	A reduction in depression levels was observed in the intervention group.	Yes: 9/13; no: 2/13; unclear: 1/13; not applicable: 1/13
Menezes et al.^19^	Randomized controlled trial	Modified Ashworth Scale (MAS)	Experimental: 11; control: 11	25 min	An improvement in motor skill levels was observed in the intervention group.	Yes: 9/13; no: 2/13; unclear: 1/13; not applicable: 1/13
Figlewski et al.^20^	Randomized controlled trial	WMFT-FAS	Experimental: 22; control: 22	30 min	An improvement in motor skill levels was observed in the intervention group.	Yes: 13/13
Huang et al.^21^	Randomized controlled trial	WMFT-FAS	Experimental: 13; control: 11	20 min	An improvement in motor skill levels was observed in the intervention group.	Yes: 13/13
Wong et al.^22^	Randomized controlled trial	Fugl-Meyer Assessment (FMA)	Experimental: 12; control: 12	20 min	An improvement in motor skill levels was observed in the intervention group.	Yes: 13/13
Kim^23^	Randomized controlled trial	Fugl-Meyer Assessment (FMA)	Experimental: 15; control: 15	20 min	An improvement in motor skill levels was observed in the intervention group.	Yes: 13/13
Liao et al.^24^	Randomized controlled trial	Fugl-Meyer Assessment (FMA)	Experimental: 14; control: 15	30 min	An improvement in motor skill levels was observed in the intervention group.	Yes: 9/13; no: 2/13; unclear: 1/13; not applicable: 1/13
Liao et al.^25^	Randomized controlled trial	Fugl-Meyer Assessment (FMA)	Experimental: 11; control: 12	90 min	An improvement in motor skill levels was observed in the intervention group.	Yes: 9/13; no: 2/13; unclear: 1/13; not applicable: 1/13
Alisar et al.^26^	Randomized controlled trial	Fugl-Meyer Assessment (FMA)	Experimental: 16; control: 16	30 min	An improvement in motor skill levels was observed in the intervention group.	Yes: 13/13
Pires et al.^27^	Randomized controlled trial	Fugl-Meyer Assessment (FMA)	Experimental: 20; control: 20	30 min	An improvement in motor skill levels was observed in the intervention group.	Yes: 9/13; no: 2/13; unclear: 1/13; not applicable: 1/13
Kim et al.^28^	Randomized controlled trial	Functional Ambulation Scale	Experimental: 9; control: 8	10 min	An improvement in motor skill levels was observed in the intervention group.	Yes: 9/13; no: 2/13; unclear: 1/13; not applicable: 1/13

FMA: Fugl-Meyer Assessment; MAS: Modified Ashworth Scale; WMFT-FAS: Wolf Motor Function Test - Functional Ability Scale.

### Ethical considerations

As this study was based exclusively on previously published data, ethical approval and informed consent were not required.

### Methodological quality assessment

Methodological quality was independently assessed by two reviewers using the Joanna Briggs Institute critical appraisal checklist for randomized controlled trials^11,12^. The methodological quality of the included studies was categorized based on the percentage of "Yes" responses to the 13 Joanna Briggs Institute (JBI) criteria: ≥ 70% was considered low risk of bias (high quality), 50–69% as moderate risk of bias (moderate quality), and <50% as high risk of bias (low quality). No studies were found to have a high risk of bias. In this study, the evaluation results for each study are shown in Table 1 as "*Quality score*" is given as^11,12^.

### Data synthesis

Statistical analyses were conducted using Comprehensive Meta-Analysis software (Version 2). Heterogeneity was assessed using the Q statistic and Higgins’ I^2^, with values above 50% indicating substantial heterogeneity^13^. Statistical significance was set at p<0.05^14^.

## FINDINGS

The characteristics and main findings of the studies are presented in Table 1; it was determined that the studies met more than half of the items in the evidence quality assessment tool (Table 1).

Meta-analysis results on the effect of transcranial direct current stimulation on motor functional independence in patients with stroke.

The study selection process based on the PRISMA flow diagram is presented in Figure 1.

**Figure 1:**
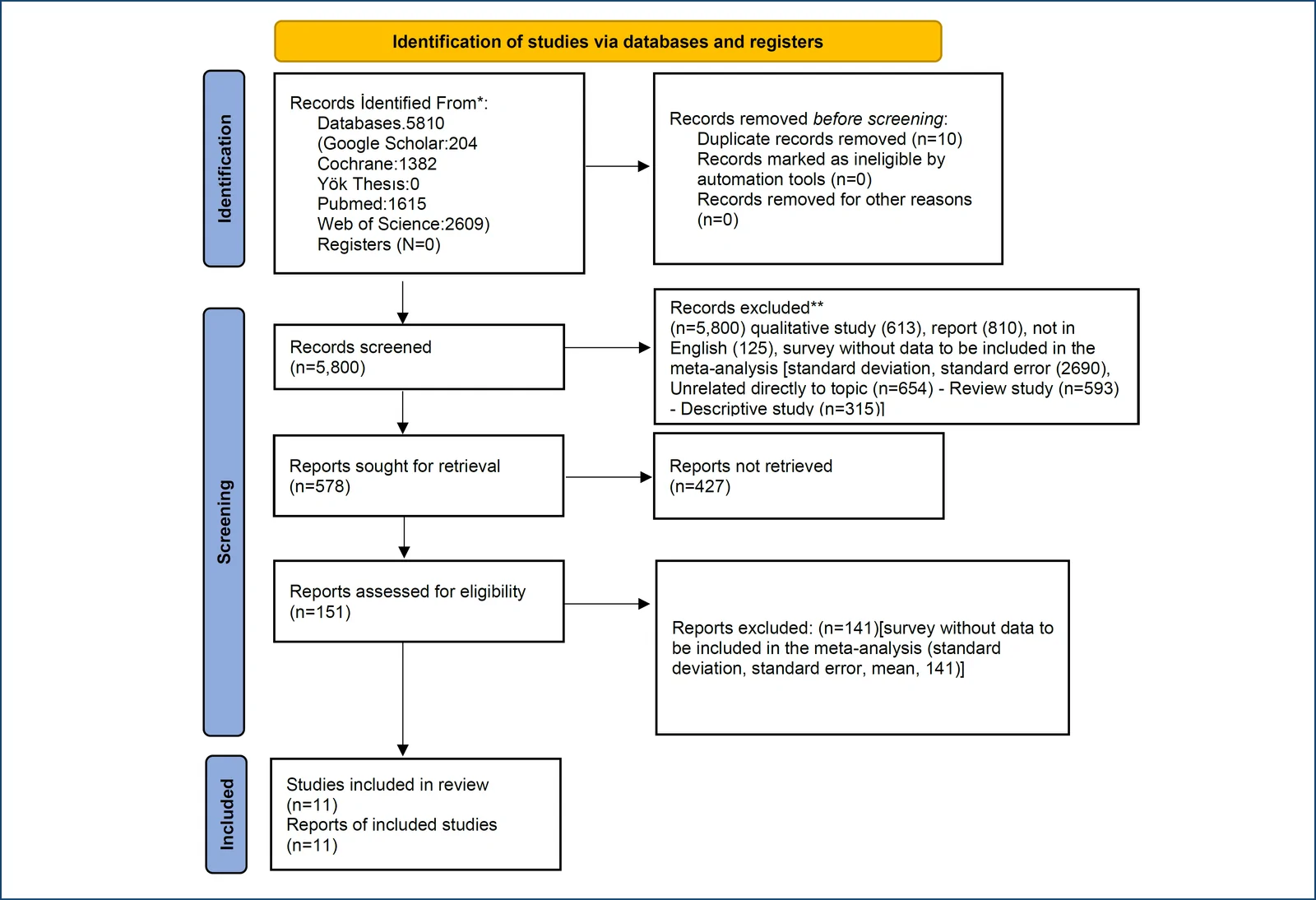
Selection of studies according to the PRISMA flow diagram. *Numbers of records identified are reported separately for each database. **Records were excluded by the reviewers; no records were excluded using automation tools.

In this study, publication bias was assessed using three methods: (a) funnel plot analysis, (b) Begg and Mazumdar's rank correlation test, and (c) Egger regression test.

Visual inspection of the funnel plot, one of the key methods for detecting publication bias, indicated that the studies were symmetrically distributed around the upper part of the funnel. This pattern suggests the absence of significant publication bias in the dataset.

Publication bias among the studies included in this dataset was further evaluated using Begg and Mazumdar's rank correlation test. According to this test, the two-tailed p-value was 0.06, indicating that publication bias was not statistically significant.

Publication bias among the studies included in this dataset was further evaluated using the Egger Regression test. According to this test, the two-tailed p-value was 0.09, indicating that publication bias was not statistically significant.

Figure 2 presents the forest plot of the 11 studies examining the effect of transcranial direct current stimulation on motor functional independence in patients with stroke, including effect sizes, standard errors, variances, and confidence intervals. Based on the findings of these studies, the meta-analysis demonstrated that transcranial direct current stimulation had a significant effect on motor functional independence in patients with stroke (SMD=-3.198; 95%CI -4.215 to -2.180; Z=-6.158; p<0.001; I^2^=89.18%) (Figure 2).

**Figure 2:**
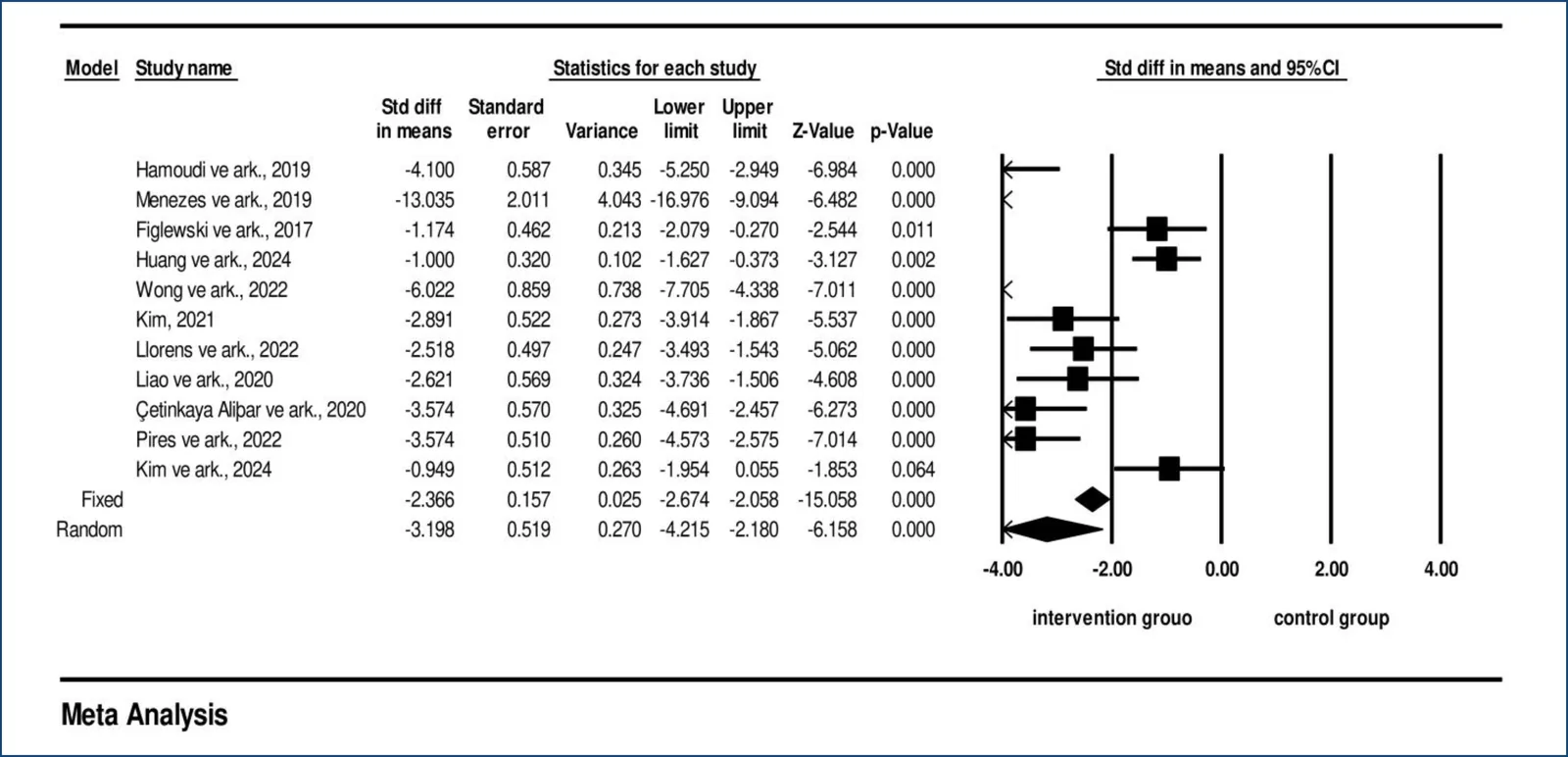
Forest plot showing the effect of transcranial direct current stimulation on motor functional independence in patients with stroke in the intervention and control groups.

The results of the moderator analysis conducted to examine the effectiveness of tDCS therapy on motor functional independence in stroke patients are presented in Table 2.

**Table 2:** Moderator analysis results regarding the effectiveness of transcranial direct current stimulation therapy on motor functional independence in stroke patients.

Moderatör (time)	Number of studies (k)	Point estimate	Standard error	95% lower limit	95% upper limit	Z	p	Qb (df)	pQb
10 min	1	-0.949	0.512	-1.954	0.055	-1.853	0.064	–	–
20 min	4	-3.409	1.033	-5.435	-1.384	-3.299	0.001	–	–
25 min	1	-13.035	2.011	-16.976	-9.094	-6.482	0.000	–	–
30 min	4	-2.687	0.589	-3.840	-1.533	-4.564	0.000	–	–
90 min	1	-2.621	0.569	-3.736	-1.506	-4.608	0.000	–	–
Groups between (Qb)	–	–	–	–	–	–	–	*37.309 (4)*	*0.000*
Overall	11	-2.360	0.302	-2.952	-1.768	-7.816	0.000	–	–

The italicized values in the between-group row of table denote the between-group heterogeneity statistic (Qb=37.309), its degrees of freedom (df=4), and the corresponding p-value. The italics identify the results of the between-group test.

However, when considering the treatment duration of tDCS used in the study as a moderator variable (p=0.000), a statistically significant difference in effect size was found (p<0.05). The study found that the duration of tDCS treatment modified the effect size on motor functional independence (p=0.00).

## DISCUSSION

This meta-analysis showed that tDCS was associated with statistically significant improvements in post-stroke motor outcomes. However, because most included studies assessed motor impairment or motor performance rather than functional independence as a direct activities-of-daily-living outcome, these findings should be interpreted as reflecting changes in motor function and functional performance rather than definitive gains in independence. In addition, the substantial heterogeneity observed across studies (I^2^=89.18%) indicates that the pooled estimate should be interpreted with caution.

The present findings are broadly consistent with previous evidence suggesting that tDCS and other non-invasive brain stimulation approaches may support post-stroke motor recovery^8,9,15-17^. Nevertheless, the interpretation of these findings requires caution because the outcomes included in the present meta-analysis were not fully homogeneous. In particular, several studies used motor impairment or motor performance measures, whereas direct measures of ADL or functional independence were limited. This distinction is important because improvements in motor impairment scores may not necessarily translate into meaningful gains in daily independence.

The high heterogeneity identified in this study likely reflects differences in stimulation parameters, including polarity, intensity, electrode montage, number of sessions, treatment duration, targeted cortical regions, adjunct rehabilitation approaches, and participant characteristics such as stroke phase and baseline motor impairment^8,9,18-28^. Variations in outcome measures may also have contributed to conceptual heterogeneity, as the included instruments assessed distinct domains, including motor impairment, motor performance, spasticity, ambulation, and functional performance^18-28^. Similar concerns have been noted in the broader post-stroke neuromodulation literature^15-17^.

Current evidence suggests that the effects of tDCS may be influenced by the rehabilitation strategies with which it is combined, including task-oriented training, physiotherapy, and other structured motor rehabilitation approaches^8,9,18-28^. Therefore, tDCS should be considered as a potential adjunct to multimodal stroke rehabilitation rather than as a standalone intervention.

From a clinical perspective, tDCS may support post-stroke motor recovery when used as an adjunct to structured rehabilitation; however, the current evidence is not sufficient to make definitive clinical recommendations. The absence of standardized stimulation protocols, small sample sizes, limited adverse event reporting, and lack of long-term follow-up restrict its implementation in routine practice. Future studies should employ larger and more homogeneous samples, standardized intervention parameters, adequate sham procedures, longer follow-up periods, and patient-centered outcome measures to clarify whether improvements in motor performance translate into meaningful functional gains in daily life^29^. Recent systematic reviews have similarly emphasized the importance of subgroup and sensitivity analyses and safety reporting when interpreting heterogeneous evidence in clinical intervention research^30,31^.

## CONCLUSION AND RECOMMENDATIONS

The findings of this study indicate that tDCS has a significant effect on motor functional independence in patients with stroke. It is recommended that future randomized controlled trials with larger sample sizes be conducted to further evaluate the effects of tDCS on various outcomes in stroke patients.

## LIMITATIONS

Some of the studies included in this systematic review and meta-analysis had small sample sizes and were conducted without blinding. These factors may reduce the strength of the evidence and limit the reliability of the findings.

## Data Availability

The datasets generated and/or analyzed during the current study are available from the corresponding author upon reasonable request.
